# UCSQ Method Applied on 3D Photogrammetry: Non-Invasive Objective Differentiation Between Synostotic and Positional Plagiocephaly

**DOI:** 10.1177/10556656221100679

**Published:** 2022-05-11

**Authors:** Sophia A.J. Kronig, Otto D.M. Kronig, Henri A. Vrooman, Léon N.A. Van Adrichem

**Affiliations:** 1 Department of Plastic and Reconstructive Surgery, 8124 University Medical Center Utrecht, The Netherlands; 2 Department of Radiology, Erasmus MC, 6993 University Medical Center Rotterdam, Rotterdam, The Netherlands

**Keywords:** classification, cranial suture, synostosis, deformational, positional, 3D photogrammetry

## Abstract

**Objective:**

Objective differentiation between unilateral coronal synostosis (UCS) and positional posterior plagiocephaly (PPP) based on 3D photogrammetry according to Utrecht Cranial Shape Quantificator (UCSQ).

**Design:**

Retrospective study.

**Setting:**

Primary craniofacial center.

**Patients, Participants:**

Thirty-two unoperated patients (17 UCS; 15 PPP) (age < 1 year).

**Interventions:**

Extraction of variables from sinusoid curves derived using UCSQ: asymmetry ratio forehead and occiput peak, ratio of gradient forehead and occiput peak, location forehead and occiput peak.

**Main Outcome Measure(s):**

Variables, derived using 3D photogrammetry, were analyzed for differentiation between UCS and PPP.

**Results:**

Frontal peak was shifted to the right side of the head in left-sided UCS (mean *x*-value 207 [192-220]), and right-sided PPP (mean *x*-value 210 [200-216]), and to the left in right-sided UCS (mean *x*-value 161 [156-166]), and left-sided PPP (mean *x*-value 150 [144-154]). Occipital peak was significantly shifted to the right side of the head in left-sided PPP (mean *x*-value 338 [336-340]) and to the left in right-sided PPP (mean *x*-value 23 [14-32]). Mean *x*-value of occipital peak was 9 (354-30) in left- and 2 (350-12) in right-sided UCS. Calculated ratio of gradient of the frontal peak is, in combination with the calculated asymmetry ratio of the frontal peak, a distinctive finding.

**Conclusions:**

UCSQ objectively captures shape of synostotic and positional plagiocephaly using 3D photogrammetry, we therefore developed a suitable method to objectively differentiate UCS from PPP using radiation-free methods.

## Introduction

The term “plagiocephaly” is derived from the Ancient Greek word πλαγιος (oblique, slanted) and was introduced by Virchow in 1851 to define the morphology of patients with skull asymmetry, due to unilateral coronal or lambdoidal suture synostosis, either congenital or acquired.^1^

Unlike unilateral coronal craniosynostosis (UCS) (congenital), positional (or deformational) plagiocephaly (acquired) is secondary to external deformational forces (positional or functional) and tends to improve with time and usually requires conservative (non-operative) treatment.^1–5^ There are 3 common types of positional skull deformities: (1) plagiocephaly (flattening on one side of the back of the head), (2) brachycephaly (equal flattening on both sides of the back of the head), and (3) scaphocephaly (equal flattening of both sides of the head [more common in premature infants]). Furthermore, a combination between positional plagiocephaly and brachycephaly can be found. In the present study, we only focus on a skewed head shape due to UCS and posterior positional plagiocephaly (PPP).

Diagnosis of both synostotic and positional plagiocephaly can be made (almost always) on clinical grounds. Differentiation between synostotic (either unilateral coronal or lambdoid synostosis) and positional plagiocephaly is essential, and in the majority of patients, anamnesis and physical examination are sufficient for this purpose.^6^ Huang et al.^7^ made a descriptive method of classification of PPP and explains how this diagnosis differs from unilateral lambdoid synostosis (posterior plagiocephaly). These criteria have been widely used in the literature. Huang et al. describe how positional plagiocephaly results in a parallelogram conformation; in contrast, unilateral lambdoid synostosis leads to a trapezoidal head shape when seen in the vertex view. Although originally described for posterior plagiocephaly, this principle has also been applied (with success) to frontal plagiocephaly secondary to UCS by many clinicians.^7,8^ The affected side of the occiput is flattened in PPP, as is the affected side of the forehead in UCS. Additionally, compensatory frontal bossing can be noted in moderate to severe cases in both deformities; ipsilateral in positional plagiocephaly and contralateral in synostotic plagiocephaly. This involvement of the forehead may progress, leading to a facial scoliosis in both diagnoses, and therefore asymmetry of the craniofacial skeleton (eg, nasal root and chin deviation, displacement of ear, superior orbital rim and palpebral fissure).^3,7,9^ It should be noted that the description is related to the severity of the PPP, as in the early stages of PPP, when the frontal bossing is not equal to the degree of occipital flattening, the cranial shape is more a trapezoid than a parallelogram.^10–12^ Additionally, Ehret et al.^13^ found that a trapezoidal head shape can be seen in rare cases of UCS with positional molding, combined anterior and posterior molding, and UCS with lambdoid synostosis. In contrast, in vertex view of patients with unilateral lambdoid synostosis, a parallelogram head shape can be found, when associated with ipsilateral PPP.^14^ Therefore, a description of a trapezoid or parallelogram head shape alone is not sufficient for correct diagnosis and further quantification.

In previous studies, we classified and quantified severity of UCS and other skull shape deformities (craniosynostosis) based on UCSQ (Utrecht Cranial Shape Quantificator).^15–17^ This is an outline-based method and captures skull shape variations. External landmarks (soft tissue landmarks, visible with the bare eye) are used to extract an outline of the skull shape (performed on CT scans), resulting in specific and characteristic curves and parameters for different subgroups of craniosynostosis. UCSQ has provided a method to diagnose and quantify the patients based on selected variables extracted from these curves. Additionally, a decisive flowchart for diagnosing the different subgroups of craniosynostosis was proposed.^17–20^ As mentioned before, UCSQ is currently performed on CT scans, however, due to the use of external landmarks and the external skull outline, UCSQ has the advantage of potential applicability to all 3D-surface rendering techniques. A promising 3D-surface method is 3D stereophotogrammetry (3D photogrammetry). Unlike conventional imaging techniques, no radiation load is used in 3D photogrammetry. It is a fast and patient-friendly method to evaluate the complete 3D morphology of the cranial shape. This imaging technique provides a less invasive method for both diagnostic and follow-up (obtaining 3D images of the cranium) purposes.

The purpose of the present study is to use the non-invasive and radiation-free imaging technique of 3D photogrammetry to extract specific parameters for the diagnosis of positional posterior plagiocephaly based on the UCSQ method. Additionally, the aim is to objectively distinguish between unilateral coronal synostosis and positional posterior plagiocephaly based on parameters of the forehead and/or occiput extracted according to the UCSQ method. The diagnostic flowchart will be adjusted accordingly.

## Material and Methods

### Patients

For the purposes of the current study, we included pre-operative 3D photos of the head of children (age < 1 year) with CT confirmed nonsyndromic UCS and children (age < 1 year) with PPP. During the 3D photogrammetry, the children wore caps, in order to minimalize loss of data due to hair growth. Diagnosis of PPP was clinically established. During medical history taking the following signs indicate a positional skull shape deformity: not present at birth, onset in the first 3 months following birth and presence of a preferred sleep position. During physical examination in a patient with PPP, the following sign can be found: parallelogram from vertex view, displacement of the entire orbit (not solely the cranial part), open anterior fontanelle (diamond-shaped fontanelle), and normal skull circumference.^8,21^

Any subjects with additional synostosis, other craniofacial abnormality, or cranial surgery prior to the first available 3D photo were excluded. 3D photos with (cranial or facial) artifacts were excluded.

### UCSQ and Resulting Curves

3D photogrammetry was performed using the 3dMD Head system (3dMD Inc). This 3D imaging system uses structured light and stereophotogrammetry. The system is set up with 5 modular units of each 3 machine vision cameras and a flash system synchronized in a single capture, resulting in a 360-degree full head capture documenting the size and shape of the patient's craniofacial complex and cranial geometry. A continuous 3D polygon surface mesh with a single *x*, *y*, *z* coordinate system from all synchronized stereo pairs is automatically generated. No stitching of the images is required (http://www.3dmd.com). The capture speed of the 3dMD system is  ± 1.5 milliseconds.

**Figure 1.**
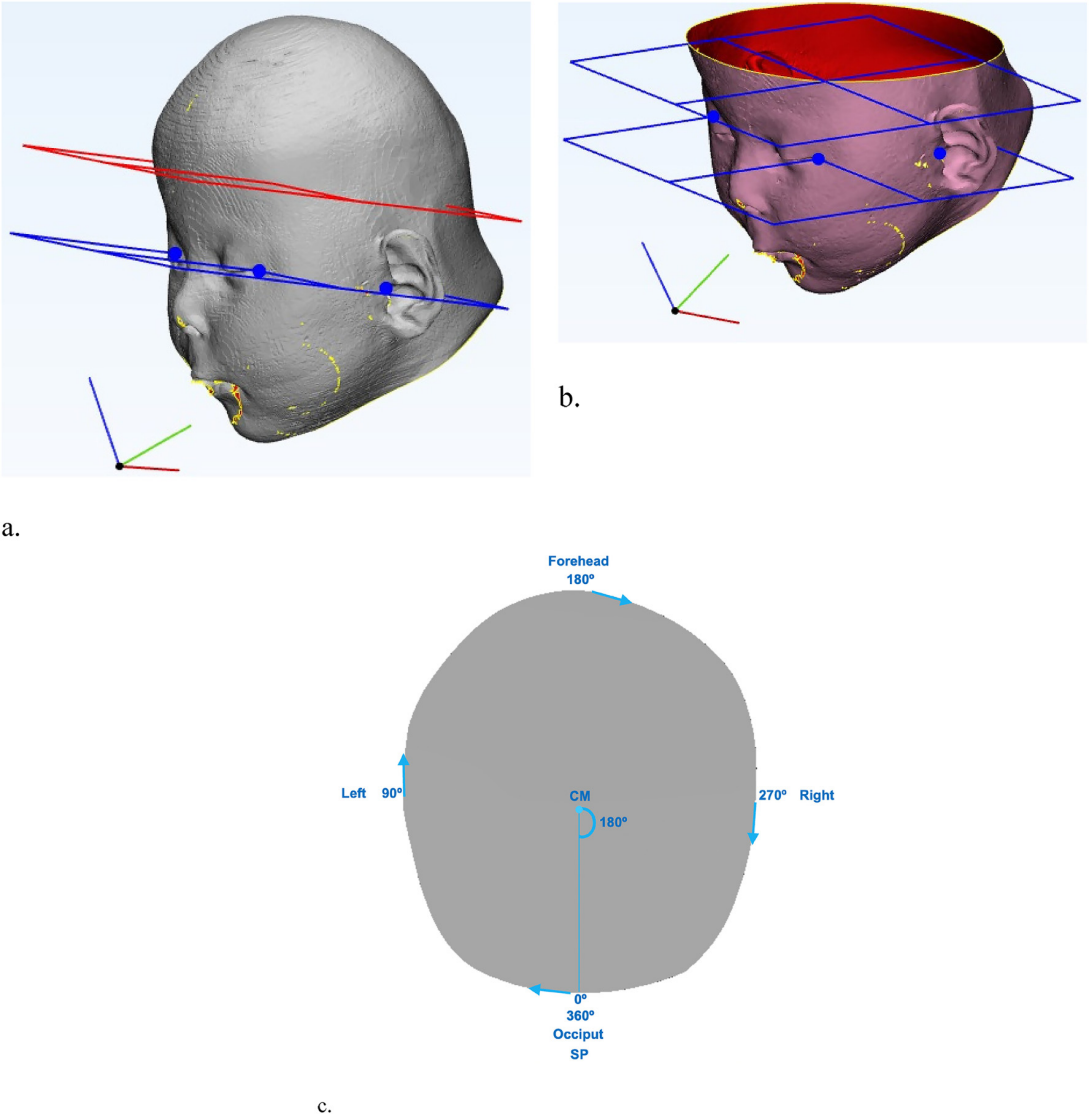
Base plane and plane 4 cm superior of and parallel to base plane; blue dots indicate placement of landmarks (left and right exocanthion, and left porion [right porion in left-sided anterior plagiocephaly]). (a) Before slicing of the plane. (b) After slicing of the plane. (c) Visualization of the starting point and the direction of the curve.

Abbreviations: CM, center of mass; SP, starting point. Arrows indicate direction of resulting curve.

The software program 3-Matic (13.0, Materialise) was used to import and analyze the 3dMD photos. As described in our previous study, 3 external landmarks are used to create a base plane in 3-Matic.^15^ The following 3 landmarks are placed: left and right exocanthion, left porion (right porion in left-sided anterior plagiocephaly) (Figure 1). Using the 3 landmarks a Datum Plane is created, this plane is duplicated and then shifted (exactly parallel to the base plane) 4 cm superiorly (Figure 1).

**Figure 2.**
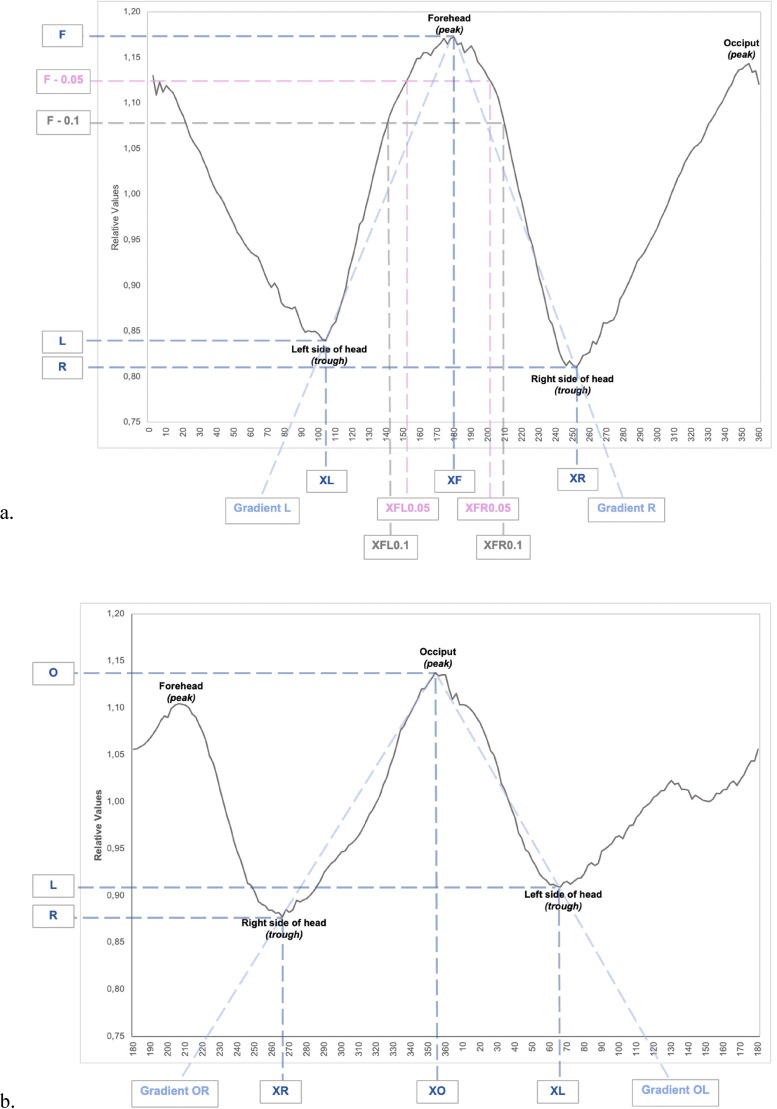
Resulting sinusoid curves; the different variables are marked. (a) Focused on the forehead. (b) Focused on the occiput.

We used the UCSQ in order to create sinusoid curves of the coupes (planes) of the included patients (Figure 2).^15^ It should be noted that, in contrast to our previous studies on CT scans, the coupes following 3D photogrammetry are analyzed from above (cranially). This results in a change in the curves; where left (first) trough now represents the left side of the head and the second trough now represents the right side of the head (Figure 2). The curve (focused on the forehead) starts at the occiput and follows the skull outline clockwise and stops where it started (at the occiput). Additionally, curves focused on the occiput were created. In these curves, the *x*-values range from 180° to 179°, compared to 0° to 360° in the forehead focused curves; this curve starts at the forehead and also follows the skull outline clockwise and stops where it started.

### Variables

Each different type of skull shape deformity results in a specific and recognizable skull shape and therefore a specific pattern of the curve, as found in our previous study.^16^ The resulting curves were analyzed, different aspects were measured and calculated, as shown in Figure 2, and Table 1. For example, the location of both the peak of the forehead and the occiput in degrees were determined.

**Table 1.** Extracted and Calculated Variables From Curve.

Extracted variable	Abbreviation	Extracted variable	Abbreviation
Maximum value of forehead peak (*y*-value)	F	*x*-value (in degrees) of the maximum forehead value	XF
Maximum value of occiput peak (*y*-value)	O	*x*-value (in degrees) of the maximum forehead value	XO
Minimum value (*y*-value) of left side of head (trough)	L	Minimum value (*y*-value) of right side of head (trough)	R
*x*-value (in degrees) for maximum forehead minus 0.1 (F−0.1) on left side of head	XFL0.1	*x*-value (in degrees) for maximum forehead minus 0.1 (F−0.1) on right side of head	XFR0.1
*x*-value (in degrees) for maximum forehead minus 0.05 (F−0.05) on left side of head	XFL0.05	*x*-value (in degrees) for maximum forehead minus 0.05 (F−0.05) on right side of head	XFR0.05
*x*-value (in degrees) of minimum value of width on left side of head	XL	*x*-value (in degrees) of minimum value of width on right side of head	XR
*Calculated variable*	*Formula*	*Calculated variable*	*Formula*
Width of frontal peak ratio	(XFR0.1−XFL0.1) /(F−0.1)	Asymmetry ratio of frontal peak ^a^	(XF−XL)/(XR−XF)
Asymmetry ratio of occipital peak ^b^	(XO−XR)/(XL−XO)	Width of frontal peak at F-0.05	XFR0.05 - XFL0.05
Vertical rise (ΔY)	- F-R - F-L - O-R - O-L	Horizontal run (ΔX) ^c^	- XR-XF - XF-XL - XO-XR - XL-XO
Gradient forehead	Δ*Y* / Δ*X*: Left side forehead peak (Gradient L) - (F−L)/(XF−XL) Right side forehead peak (Gradient R): - (F−R)/(XR−XF)	Gradient occiput ^c^	Δ*Y* / Δ*X*: Left side occiput peak (Gradient OL): - (O−L)/(XL−XO) Right side occiput peak (Gradient OR): - (O−R)/(XO−XR)
Ratio of gradient of forehead peak in: - Left-sided UCS - Right-sided PPP	Gradient L / Gradient R	Ratio of gradient of forehead peak in: - Right-sided UCS - Left-sided PPP	Gradient R / Gradient L
Ratio of gradient of occiput peak in: - Right-sided UCS - Right-sided PPP	Gradient OR / Gradient OL	Ratio of gradient of occiput peak in: - Left-sided UCS - Left-sided PPP	Gradient OL / Gradient OR

- XO more than 360° (ie, low value, right of 360° in occiput curve): only in (XO−XR), the XO value is 360 + XO.

- XO less than 360° (left side in occiput curve of 360°): in (XL−XO), the XL value is 360 + XL.

- XO more than 360° (ie, low value, right of 360° in occiput curve): only in (XO−XR), the XO value is 360 + XO.

- XO less than 360° (left side in occiput curve of 360°): in (XL−XO), the XL value is 360 + XL.

For both the forehead and the occiput, we considered the curve between the trough and peak as a straight line for the purposes of the calculation of gradient (slope) of this line (Figure 2). Regarding the forehead, the troughs XL, L and XR, R are used (representing the *x*- and *y*-values of these troughs) and the peak is XF, F (representing the *x*- and *y*-value of the forehead). Regarding the occiput the troughs XR, R and XL, L are used (representing the *x*- and *y*-values of these troughs) and the peak is XO, O (representing the *x*- and *y*-value of the occiput).

This gradient (slope) can be calculated with the general formula: gradient = vertical rise/horizontal run. Table 1 shows the specific formula using variables extracted from our created curve. It should be noted that the ratio of gradient of the occiput peak was calculated as Gradient OR / Gradient OL in right-sided UCS and PPP (in contrast to Gradient OL / Gradient OR in left-sided UCS and PPP). Furthermore, in these calculations of the gradients of the occiput peak, attention was paid to the *x*-value of the occiput peak (see footnotes of Tables 1 and 2).

**Table 2.** Extracted and Calculated Variables From Curve for Different Craniosynostosis Subgroups.

	Left-sided anterior plagiocephaly (*N* *=* *10*)	Right-sided anterior plagiocephaly (*N* *=* *7*)	Left-sided posterior positional plagiocephaly (*N* *=* *4*)	Right-sided posterior positional plagiocephaly (*N* *=* *11*)
**F** *(mean (min.-max.))*	1.11 (1.08-1.13)	1.11 (1.06-1.15)	1.11 (1.07-1.16)	1.09 (1.07-1.13)
**XF** *(mean (min.-max.))*	207 (192-220)	161 (156-166)	150 (144-154)	210 (200-216)
**O** *(mean (min.-max.))*	1.11 (1.06-1.14)	1.09 (1.05-1.14)	1.13 (1.09-1.18)	1.11 (1.07-1.18)
**XO** ^a^ *(mean (range min.-max.))*	9 (354 - 30)	2 (350 - 12)	338 (336-340)	23 (14-32)
**L** *(mean (min.-max.))*	0.91 (0.87-0.95)	0.89 (0.87-0.91)	0.89 (0.85-0.93)	0.88 (0.83-0.92)
**R** *(mean (min.-max.))*	0.90 (0.87-0.94)	0.92 (0.89-0.94)	0.86 (0.81-0.89)	0.92 (0.89-0.95)
**XL** *(mean (min.-max.))*	94 (66-122)	90 (66-104)	69 (58-92)	113 (98-120)
**XR** *(mean (min.-max.))*	272 (246-302)	270 (250-280)	248 (240-260)	297 (276-312)
**Width of frontal peak ratio** *(mean (min.-max.))*	79 (62-96)	87 (59-127)	92 (72-114)	95 (76-117)
**Asymmetry ratio of frontal peak** *(mean (min.-max.))*	1.8 (1.2-2.5)	0.7 (0.5-0.8)	0.8 (0.7-0.9)	1.1 (0.9-1.4)
**Asymmetry ratio of occipital peak** ^b^ *(mean (min.-max.))*	1.2 (0.6-2.0)	1.0 (0.9-1.2)	1.0 (0.8-1.2)	1.0 (0.7-1.6)
**Ratio of gradient frontal peak (slope)** *(mean (min.-max.))*	0.6 (0.4-0.9)	0.6 (0.4-0.7)	0.9 (0.8-1.0)	1.1 (1.0-1.4)
**Ratio of gradient occiput peak (slope)** *(mean (min.-max.))*	1.1 (0.7-1.7)	0.8 (0.6-1.0)	0.9 (0.6-1.1)	0.9 (0.5-1.2)

^a^ Values of XO are around 360°, this can be either less than 360° (left side in curve of 360°; eg, “358”) or more (right side in curve of 360°; eg, “2”). For calculation of the mean value of XO, we added for each subject with a value on the right side of 360° this value to 360. Following calculation, when the mean value was more than 360°, we subtracted 360, this value was noted in this table. Minimum and maximum values indicate the range of *x*-values around 360°.

^b^ Please see footnotes in Table 1.

In case of a value of XO lower than 360° (left side in curve of 360°): in (XL−XO), the XL value was 360° + XL (resulting in [360° + XL−XO]).

The asymmetry ratio of the frontal peak is calculated and shown in Table 1. An asymmetry ratio of  ≤ 0.8 was used to describe a peak shifted to the right side of the head and  ≥ 1.2 for a peak shifted to the left side of the head, a ratio of 0.8 to 1.2 equals no significant shifting of the forehead peak.

Additionally, shifting of the occiput peak was assessed by calculation of the asymmetry ratio of occiput peak. In this calculation, attention was paid to the *x*-value of the occiput peak. For calculation of the asymmetry ratio of occipital peak, we used the following rules to obtain the correct values following calculation: (1) If the value of XO was more than 360° (i.e., low value, right of 360° in curve): only in (XO−XR), the XO value was 360° + XO. (2) If the value of XO was lower than 360° (left side in curve of 360°): in (XL−XO), the XL value was 360° + XL.

### Severity of UCS and PPP

Severity of the condition in patients with UCS was determined by the UCSQ-based calculation: (asymmetry ratio of frontal peak − 1.067) ×  − 0.23 + (ratio of gradient − 0.90) × 0.57. In this calculation, asymmetry ratio of frontal peak is calculated as follows: affected side / unaffected side (left-sided UCS: (XF−XL)/(XR−XF); right-sided UCS: (XR−XF)/(XF−XL)). The following cutoff values were used for each subgroup of severity: mild ≥ − 0.1, moderate  − 0.1 to  − 0.5, severe ≤ − 0.5.^22^

Severity of PPP in the included patients was determined by plagiocephalometry (PCM). PCM is a non-invasive instrument to assess and quantify the asymmetry of the skull in patients with positional plagiocephaly. Originally, PCM is performed with a strip of thermoplastic material positioned around the infant's head at the widest transverse circumference. The following landmarks are originally located on the thermoplastic ring; both ears, nose, and the middle of the posterior circumferential distance between the left and right ear. The upper side of the ring is copied on paper. Lines are drawn on the paper copy and measured, by which the degree of asymmetry can simply be determined by calculating the differences between the lengths of the left and right lines.^23,24^

In the present study, the coupes created with UCSQ were used for PCM in patients with PPP. The nose landmark was considered at 180° and the “posterior circumferential distance between the left and right ear” at 0°; therefore, the AP (anteroposterior) line is the *y*-value at 180° + *y*-value at 0°. For the diameter difference, we calculated the oblique diameter difference index (ODDI). Oblique diameter left (ODL) and oblique diameter right (ODR) lines are drawn from points located 40° either side of the anteroposterior (AP) line. In the present study using UCSQ, ODL was calculated as: *y*-value at 40° + *y*-value at 220°; ODR was calculated as: *y*-value at 140° + *y*-value at 320°. ODDI was calculated as follows: ratio between the ODL and the ODR as the longest/shortest diameter × 100%. An ODDI of more than 104% illustrates obvious clinical asymmetry of the skull. Furthermore, for transversal shape and proportion of the skull, we calculated the cranio proportional index (CPI): ratio between the sinistra-dextra (SD; in the present study: *y*-value L +* y*-value R) and the anteroposterior (AP) is calculated as SD / AP × 100%.^23,24^ CPI is the same principle as cranial index (CI), a CI that is 85% or greater is considered deviant and corresponds to a brachycephalic skull.^25^

### Decisive Flowchart

Extracted values for both synostotic and positional plagiocephaly were compared in order to establish distinctive parameters for diagnosis and run through the previously CT-validated diagnostic flowchart.^16^ Following, based on the extracted and calculated values, a new diagnostic flowchart was established.

### Statistical Analysis

Statistical analyses were performed using the Statistical Package for the Social Sciences for Windows (Version 21, SPSS Inc). Descriptive statistics were calculated. Unpaired *t*-test was used to compare the age of the patients with UCS and PPP. Statistical significance was set at *P* < .05.

## Results

### Demographics

We included pre-operative 3D photos of the head of 17 children (age < 1 year) with CT-confirmed nonsyndromic UCS and 15 children (age < 1 year) with PPP.

Mean age of the included patients with UCS was 6.9 months (1-11 months), there were 8 boys and 9 girls. Ten patients had left-sided UCS and 7 patients had right-sided UCS.

Mean age of the included patients with PPP was 5.3 months (1-9 months), there were 14 boys and 1 girl. Four patients had left-sided PPP and 11 patients had right-sided PPP.

No statistical significant difference was found between the age of the included patients with UCS compared and the age of included patients with PPP (*P* > .05).

### Extracted and Calculated Variables

**Figure 3.**
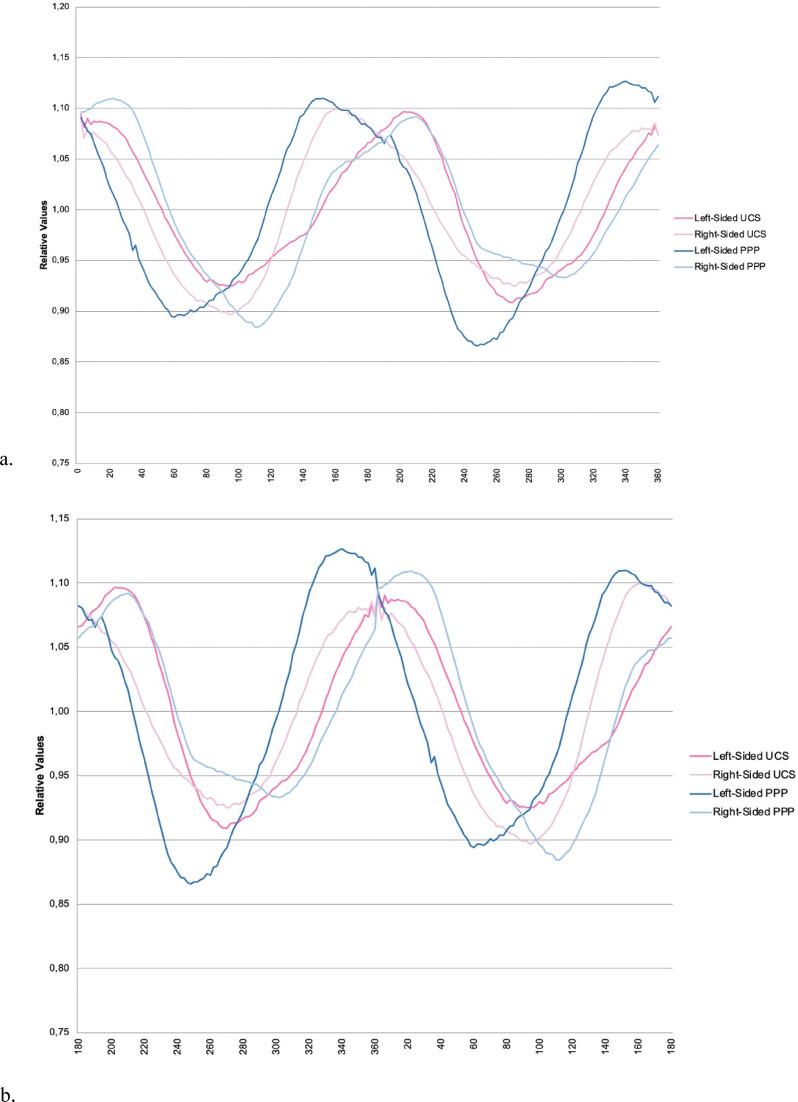
Mean curves of UCS and PPP. (a) Focused on the forehead. (b) Focused on the occiput.

Sinusoid curves are created for each patient; Figure 3a shows the mean curves focusing on the forehead of each subgroup. Figure 3b shows the mean curves of each subgroup focusing on the occiput. The extracted and calculated variables are presented in Table 2 (Table 1 shows the explanation of these variables).

### Severity of UCS and PPP

UCS was mild in 18% (3/17; 2 left- and 1 right-sided UCS), moderate in 53% (9/17; 4 left- and 5 right-sided UCS), and severe in 29% (5/17; 4 left- and 1 right-sided UCS) of the included patients according to the UCSQ quantification method.

According to PCM applied to the included patients with PPP, mean ODDI in left-sided PPP was 115% (110%-126%), and in right-sided PPP 112% (105%-122%). Mean CPI in left-sided PPP was 80% (73%-84%), and in right-sided PPP 85% (80%-91%).

### Flowchart

**Figure 4.**
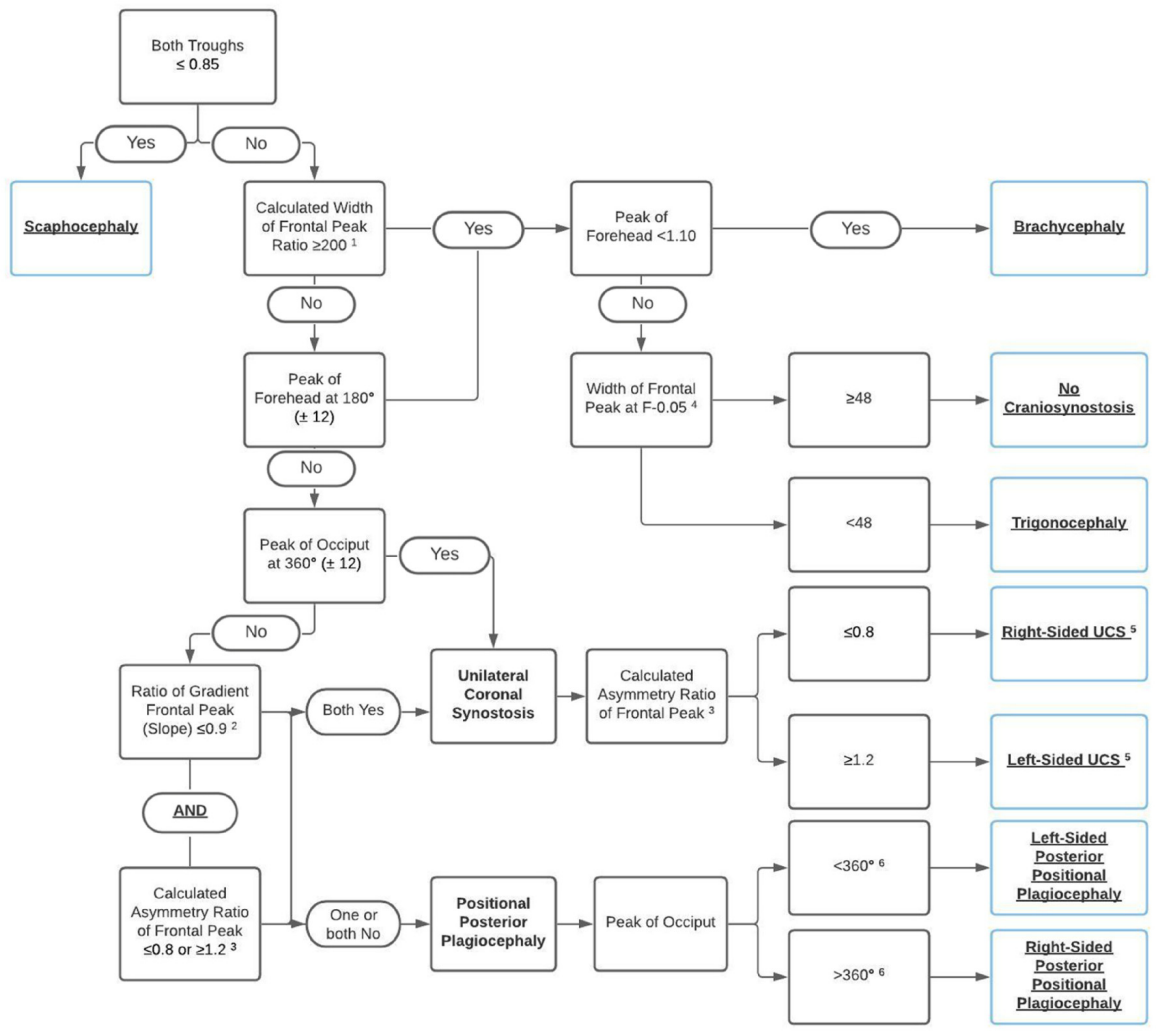
Approach to diagnosis of UCS and PPP on 3D photogrammetry.

^1^ Width of frontal peak ratio: (XFL0.1−XFR0.1) / (F−0.1)

^2^ Ratio of gradient frontal peak (slope): gradient affected side/gradient unaffected side or gradient right side/gradient left side

^3^ Asymmetry ratio of frontal peak: (XF−XR)/(XL−XF)

^4^ Width of frontal peak at F−0.05: (XFL0.05−XFR0.05)

^5^ Note that right- and left-sided UCS are switched in the flowchart for 3D photogrammetry in comparison with the flowchart for CT scanning

^6^ XO more than 360°: low value, right of 360° in curve (eg, 20°); XO less than 360°: left side in curve of 360° (eg, 340°).

Figure 4 shows the adjusted and newly proposed diagnostic flowchart based on the extracted and calculated variables for both synostotic and positional plagiocephaly. When using the proposed flowchart for the 32 included patients for validation, each of the patients is diagnosed correctly based on the different steps in the flowchart.

## Discussion

In the present study, we used the UCSQ method on 3D photogrammetry in order to objectively differentiate between UCS and PPP, based on the following extracted specific parameters: location of the occiput peak in the curve, ratio of gradient, and calculated asymmetry ratio of frontal peak. Currently, all patients with (suspected) UCS receive CT scanning for confirmation of the diagnosis, as this is according to the current Dutch guideline “Treatment and care for craniosynostosis.” Patients with PPP do not receive CT scanning.^21^

Many different methods for classifying and quantifying craniofacial asymmetry (both UCS and PPP) have been reported in literature, including visual assessment,^3,7,26,27^ tape measure,^28^ calipers,^14,29,30^ and flexible strips.^23,25,31^ Nonetheless, difficulties with these methods are the incomplete capturing of the whole skull, the subjectivity, and the need of CT scanning, and therefore radiation load and possible need for sedation in children. A promising and already used radiation-free imaging method is 3D photogrammetry plagiocephaly assessment, using digital computation of ODDI and CPI.^24,25^ Additionally, several other (automated) methods of assessment and measurement of skull shape and/or volume using 3D photogrammetry are present.^32–36^ Furthermore, De Jong et al.^37^ demonstrated that 3D stereophotogrammetry combined with deep learning (a modern machine learning technique) can provide a basis to accurately classify cranial shapes of healthy controls, scaphocephaly patients, trigonocephaly patients, and anterior plagiocephaly patients, and therefore used it as a diagnostic tool.

With the previously mentioned limitations in mind, UCSQ was created and validated for, among other diagnoses, UCS.^15–17^ In our previous study, a good correlation between UCSQ and commonly used visual scores for UCS was found, indicating that the visual aspects of asymmetry can be (objectively) put into numbers of severity using UCSQ.^17^ Of the included patients with UCS in the current study, severity ranged from mild (18%) and moderate (53%) to severe (29%) according to UCSQ. Initially, UCSQ was validated on CT scans, but is in the current study applied on 3D photogrammetry. This is a fast and patient-friendly method to evaluate the complete 3D morphology of the cranial shape, without either radiation load or need for sedation.

In the present study, PCM was used to indicate severity of PPP. PCM is a non-invasive method to quantify skull asymmetry in patients with PPP, taking several aspects of the (asymmetric) skull into account (ear and nose position, local flattening of the skull, diameter difference, transversal shape, and proportion of the skull).^24^ The indices ODDI and CPI provide additional information about the amount of asymmetry and proportion in the growing skull. In PCM, an ODDI of more than 104% illustrates obvious clinical asymmetry of the skull and is, therefore, clinically relevant and corrected for age and growth. Mean calculated ODDI in the current study was 115% in left-sided PPP and 112% in right-sided PPP, indicating that all included patients with PPP had an obvious clinical asymmetry of the skull. CPI is the same principle as CI, a CI that is 85% or greater is considered deviant and corresponds to a brachycephalic skull.^25^ Mean CPI was 80% (73%-84%) in left-sided PPP and 85% (80%-91%) in right-sided PPP, indicating no brachycephalic skull was found in patients with left-sided PPP, however 7 included patients with right-sided PPP had a brachycephalic skull. In the included patients with PPP and a brachycephalic skull, the PPP component was more prominent than the brachycephalic component and was therefore initially not mentioned in the medical records. It should be noted that PCM is not developed and validated for quantification of severity of UCS. Additionally, due to the relatively small sample size, we cannot make strong conclusions on the found differences in presence of brachycephaly between left- and right-sided PPP, as these differences may be due to chance.

In our previous studies, we found that the most distinctive variables for UCS were the asymmetry ratio of the frontal peak and the ratio of gradient of legs of forehead peak.^15–17^ Additionally, we determined if the forehead peak was located at 180° (± 12). This value of 12° corresponds to the used value of asymmetry of >3.5% in the CVAI (3.5% of 360° corresponds to a value of 12.6), which shows significantly asymmetrical values of the head in patients with plagiocephaly.^25^ These aforementioned variables are a reflection of the asymmetry of the forehead. In the present study, in addition to these variables of the forehead, we calculated the asymmetry ratio of the occiput peak, the ratio of gradient of legs of occiput peak and we determined if the occiput peak was located at 360° (± 12).

None of the included patients with either UCS or PPP had a peak of forehead within 180° ± 12, indicating a shifting of the forehead in all included patients. Furthermore, none of the included patients with PPP had an occiput peak within the 360° ± 12 range, indicating a shifting of the occiput. However, 3 of the 17 included patients with UCS (all left-sided) had an occiput peak outside the 360° ± 12 range. After further examination of the 3D photos of these 3 patients, we can see that these patients have an accompanying positional deformity of the occiput. Therefore, if the peak of the occiput is outside the 360° ± 12 range, a positional deformity of the occiput should be considered. However, due to the relatively small sample size, we cannot make strong conclusions on the found differences in presence of accompanying positional deformity between left- and right-sided UCS, as these differences may be due to chance.

As stated before, the shape of the PPP skull in vertex view is a parallelogram.^7^ This parallelogram shape is supported by our results. In all included patients with right-sided PPP, the forehead peak is shifted more than 12° right of 180° (this means to the right side of the head), additionally in all these patients the occiput peak is shifted more than 12° right of 360° (this means to the left side of the head). Combining these 2 findings, this reflects a parallelogram-shaped skull. On the contrary, in all included patients with left-sided PPP, the forehead peak is shifted more than 12° left of 180° (this means to the left side of the head), additionally in all these patients, the occiput peak is shifted more than 12° left of 360° (this means to the right side of the head); also resulting in a parallelogram. The combination of these 2 findings is a step in the proposed decisive flowchart for the diagnosis of PPP. Additionally, the position of the occiput peak (left or right of 360°) determines the (“affected”) side of the PPP in our flowchart.

The calculated ratio of gradient of the frontal peak is, in combination with the calculated asymmetry ratio of the frontal peak, a distinctive finding. When both are relatively removed from 1.0 (ie,  ≤ 0.9 for ratio of gradient of frontal peak and either  ≤ 0.8 or  ≥ 1.2 for asymmetry ratio of frontal peak), this indicates an asymmetrical frontal peak, due to UCS. In patients with right-sided PPP, we found that the ratio of gradient of frontal peak was 1.0 in 5 of 15 included patients, additionally, in 2 of 15 patients this ratio was 1.1. This indicates an asymmetric peak of the forehead, most likely due to the parallelogram-shaped head caused by PPP. Additionally, the asymmetry ratio of frontal peak was within the 0.8 to 1.2 range in 8 of 15 included patients with PPP.

In order to further analyze the occiput, we calculated ratio of gradient of the occiput peak and the asymmetry ratio of the occiput peak. These calculated variables did not appear to be distinctive between UCS and PPP. For the patient groups (UCS and PPP; both left- and right-sided), the calculated asymmetry values were mostly around 1.0; however, some exceptions (outliers) were found. The mean calculated ratio of gradient of the occiput peak in patients with left-sided UCS 1.1 (0.7-1.7) and in right-sided UCS the mean was 0.8 (0.6-1.0). The mean calculated ratio of gradient of the occiput peak in patients with left-sided PPP 0.9 (0.6-1.1) and in right-sided PPP the mean was 0.9 (0.5-1.2). Furthermore, the mean calculated asymmetry ratio of occiput peak was 1.2 (0.6-2.0) and 1.0 (0.9-1.2) in patients with left- and right-sided UCS, respectively, the mean value was 1.0 (0.8-1.2) and 1.0 (0.7-1.6) in patients with left- and right-sided PPP, respectively.

Several limitations should be considered when interpreting the results. We used data from only one craniofacial center, resulting in an apparent relatively small patient group. However, we included a homogeneous group of patients, with regard to age and preoperative status. A study on a greater cohort could highlight the benefits of UCSQ and determine the generalizability to other populations. Secondly, this study would include the general drawback of any retrospective study.

In the present study, we used 3D photogrammetry to obtain the coupes of the skull. Because no potentially harmful ionizing radiation or sedation is required, 3D photogrammetry is an ideal technique to acquire a 3D image of the cranial shape for diagnosis and during follow-up. Furthermore, future research is necessary in order to establish an objective quantification method for the severity of PPP based on UCSQ on 3D photogrammetry. Consequently, this technique can be used during follow-up and evaluation of both surgical and non-surgical treatment.

Utrecht Cranial Shape Quantifier on 3D photogrammetry is available to objectively differentiate between UCS and PPP with the use of distinctive features of UCS and PPP (location of the occiput peak in the curve, ratio of gradient, and calculated asymmetry ratio of frontal peak), which has the advantages of capturing the whole skull shape, no radiation load, and no need for sedation. However, future research with more included patients is needed for further validation of the implemented flowchart and methods.
